# Non-Surgical Lower Face Contouring in an Indian Patient: A Case Study

**DOI:** 10.7759/cureus.35508

**Published:** 2023-02-26

**Authors:** Reema Tebak Arora, Stuti Arora, Isha Kaushik, Chetan Patil

**Affiliations:** 1 Aesthetic Dermatology, The Face Clinic, New Delhi, IND; 2 Dermatology, Kasturba Medical College, Manipal, IND; 3 Medicine, Asia-Pacific Menarini India Pvt Ltd, Mumbai, IND; 4 Internal Medicine, Asia-Pacific Menarini India Pvt Ltd, Mumbai, IND

**Keywords:** hifu, ageing, lower face, filler, pcla threads

## Abstract

A 31-year-old Indian female patient presented with a ptotic face with signs of lower face ageing. She was concerned about sagging, an older look, and blunting of the jawline. She wished to have a more oval and narrow face contour. After the evaluation of the patient, we decided to perform a sequential treatment. Initially, the lower face was debulked with high-intensity focused ultrasound (HIFU). Following that, the jawline reshaping (JR) and malar reshaping (MR) techniques were performed with Definisse double needle 12 cm poly-ε-caprolactone-co-l-lactic acid (PCLA) threads. Lower-face hyaluronic acid (HA) filler injections were given for final contouring. Global Aesthetic Improvement Scale (GAIS) and subject level of satisfaction scores showed consistent improvement with the sequential procedures and at the six-month follow-up. Overall, the treatment procedures were uneventful and without any major adverse events. The current case of an Indian patient with a ptotic face and evident signs of lower face ageing showed improvement with a combination of procedures including Definisse threads.

## Introduction

The mandibular contour and its shape are the decisive factors to determine the attractiveness of a female face. This is the predominant reason for lower face treatments being often requested in aesthetic practices, and the prevalence of this procedure is gradually burgeoning. Lower face heaviness and loss of jawline definition are common concerns even in young patients of Indian ethnicity. Irrespective of a normal BMI, the lower face bulk in itself is seen as overweight, fat, and, bigger in perspective.

Facial ageing is a biological process where all the layers of the face get affected. The loss of alveolar support and depletion of deep and superficial fat pads cause the lower face to lose its structure and definition affecting the jawline, chin, jowls, and lips. With ageing, the lower face exhibits pronunciation of jowls giving the jawline an apple-shaped appearance. Due to the loss of underlying support, excess skin accumulates. A volume deficit is observed as the chin loses fat, resulting in a loss of contour and a wider appearance. The lips undergo volume deficit changes as well, making them thinner and wider than before. Due to the repeated action of muscles, depletion of fat, and the effect of gravity on excess skin, there is a typical turkey neck appearance [1-3].

For the ageing face, various medical interventions like hormone therapy, platelet-rich plasma, lasers, facial fillers, etc. as well as surgical interventions like chin augmentation, midface lift, and thread lift are currently available [4-5]. Amongst them, the biocompatible and hydrolyzable poly(ε-caprolactone-co-L-lactic acid) (PCLA) is of great interest for medical applications. Poly(ε-caprolactone) (PCL) is a semi-crystalline material with rubbery properties, while poly (L-lactic acid) (PLA) is a crystalline and hard material. PCLA can be formulated to decompose over a period of several weeks [4-6]. The current study aims to provide insights into the evaluation and management of ageing, which includes PCLA threads, hyaluronic acid (HA) fillers, and other modalities used to address lower face ageing.

## Case presentation

A 31-year-old Indian female patient presented with a ptotic face with evident signs of lower face ageing like sagging around the nasolabial fold and the oral commissure. She was concerned about sagging, an older look, and blunting of the jawline. She wished to have a more oval and narrow face contour. She had no history of diagnosed pathologies or pre-existing skin lesions. After the evaluation of the patient, we decided to perform a sequential treatment.

The lower face was debulked with high-intensity focused ultrasound (HIFU) as the first modality (day 0). The patient was treated with HIFU (720 lines full face in a three-layer approach) using 4.5, 3 mm and 1.5 mm probes. Clinical studies have shown this to be a safe and effective way to improve skin texture and contour. The effect on subcutaneous fat causes debulking, and its effect on the superficial musculoaponeurotic system (SMAS) causes the lifting of the jowls and contouring [7-9]. One month post-HIFU (day 30), jawline reshaping (JR) and malar reshaping (MR) techniques were performed with Definisse double needle 12 cm PCLA threads. Definisse double-needle threads are the latest 4th generation absorbable, monofilament, convergent, suspension barbed threads. The MR technique helps to reshape the facial frame by lifting the mid-face, whereas the JR technique lifts and contours the lower face. Two pairs of threads were used at the same sitting. One month after the Definisse threads procedure (day 60), the patient was injected with HA fillers. Four millilitres of 20 mg/ml HA were injected in the lower face-gonial angle, lateral cheek zygomatic bone, mid-cheek area and pre-auricular area for facial reshaping, with an average dose of 0.5 ml per site. This helped with the final contouring of the lower face.

Procedures were performed under complete aseptic precautions. Post the thread procedure, in order to avoid thread displacement, all patients are advised to refrain from vigorous exercises, rubbing on the treated areas, dental procedures, and excessive face and neck movements for the first three weeks. In case of severe pain, they are advised to take oral paracetamol twice daily as well as a prophylactic antibiotic. Digital photographs with a front view and 90º side profile view were taken. Patient outcomes were evaluated using the Global Aesthetic Improvement Scale (GAIS) (Table 1) [10]. The subject’s satisfaction response was evaluated on a scale of 0 to 5 (no to maximum). Also, the subject’s level of tolerability (discomfort) was evaluated on a scale of 0 to 5 (no to maximum discomfort), and the occurrence of adverse events was recorded for safety assessment.

**Table 1:** Grades of the Global Aesthetic Improvement Scale

Score	Grade	Definition
1	Worse	The appearance is worse than the original condition
2	No change	The appearance is essentially the same as the original condition
3	Improved	Obvious improvement in appearance from the initial appearance but a touch-up is indicated
4	Much improved	Marked improvement in appearance from the initial condition but not completely optimal for this subject
5	Very much improved	Optimal cosmetic results

**Figure 1:**
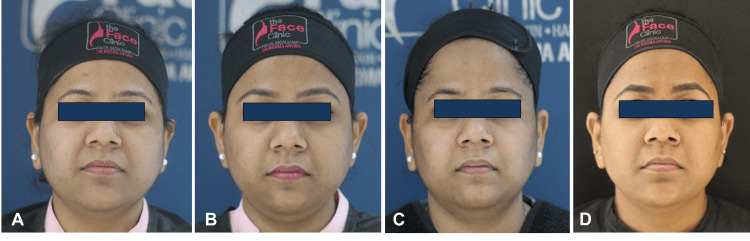
Front profile of patient; A) Day 0, photograph before HIFU; B) Day 30, after one month of HIFU and before Definisse threads JR and MR procedure; C) Day 60, after one month of Definisse threads procedure and before HA filler injections; D) Day 90, after one month of HA filler injections HIFU: high-intensity focused ultrasound; HA: hyaluronic acid; JR: jawline reshaping; MR: malar reshaping.

**Figure 2:**
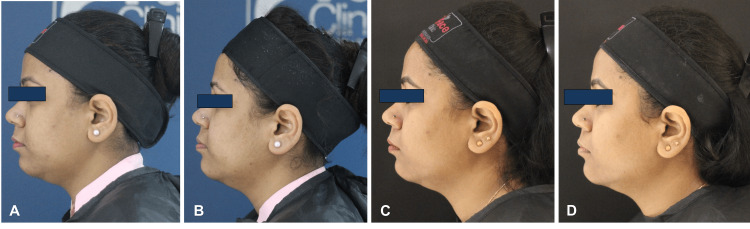
Side profile of patient; A) Day 0, photograph before HIFU; B) Day 30, after one month of HIFU and before Definisse threads JR and MR procedure; C) Day 60, after one month of Definisse threads procedure and before HA filler injections; D) Day 90, after one month of HA filler injections HIFU: high-intensity focused ultrasound; HA: hyaluronic acid; JR: jawline reshaping; MR: malar reshaping.

GAIS and subject-level satisfaction scores are reported in Table 2, showing consistent improvement with the sequential procedures and at the six-month follow-up. Subject level of tolerability for HIFU and HA fillers was 2 out of 5, while some were 3 for the threads procedure, considering thread procedures are relatively more invasive compared to the other two procedures. Clinical photographs after the one-month follow-up of each procedure are shown in Figures 1-2. There were no major adverse events; the pain post thread was minimal and the patient did not require pain killers bruising post threads resolved spontaneously and no other adverse event was noted. 

**Table 2:** Post Procedures GAIS and Subject Level of Satisfaction GAIS: Global Aesthetic Improvement Scale; HIFU: high-intensity focused ultrasound; HA: hyaluronic acid.

	Day 30 Post HIFU	Day 60 Post Definisse Threads	Day 90 Post HA fillers	Day 180 Follow-up
GAIS	2	3	4	5
Subject level of satisfaction	2	3	4	5

## Discussion

Upon discussing the patient’s initial aesthetic desire, a clinical evaluation is done with the available modalities; the decision to use a particular modality is entirely case specific. As a part of the treatment plan, HIFU therapy is prioritized due to its known effect of degradation on threads and fillers. Once the initial debulking of the face is achieved through HIFU, it is followed by threads and filler respectively. HIFU treatment takes a month for visible clinical results, thereafter, a thread lift is planned. In case the patient has lesser volume and more sag, a thread lift should be the initial step.

The thread lift procedure was done on our patient to help her midface and lower face ptosis. This procedure helps in lifting and repositioning the fat. The final step focuses on contouring and adding the desired volume and shape. The gradual increase in GAIS score emphasises the gradual improvement, as HIFU, threads, and fillers gradually integrate into the tissue leading to a cumulative show with collagen build-up. 

A thorough knowledge of the anatomy of the ageing face is required for an effective and safe surgical procedure [2]. Bioabsorbable barbed suspension double-needle threads are popular as a minimally invasive alternative for skin repositioning. As compared to surgical face lifting, these threads require less procedural and recovery time with no general anaesthesia. They confer immediate patient satisfaction, do not cause cutaneous incisions or apparent scars, and are more tolerable. These threads have great versatility for use on Asian and Caucasian facial types. They can be used to achieve nonsurgical lifts to a certain extent [4,11]. PCLA has been used as absorbable sutures for a long period and has a well-established efficacy and safety profile. Moreover, it also has a well-defined biocompatibility and degradation profile [8]. Definisse threads, when they are implanted sub-dermally, exert immediate mechanical action to lift and reposition sagging facial tissue. Over a period, its histological revitalising action stimulates fibroblasts and the synthesis of collagen, HA and elastin around the thread [4,11]. The management of age-related changes in different layers of the face has transformed due to the use of HA fillers. HA fillers have, over a period of time, become the preferred choice for soft tissue dermal corrections. It enables the creation of a smooth, uninterrupted subcutaneous layer by volumizing irregularities. The combination of Definisse threads and HA fillers can be effective in treating mild to moderate signs of ageing, where threads help in lifting the sagging skin and fillers contour the skin [11-13].

## Conclusions

Non-surgical lower face contouring with a combination of treatments in Indian patients shows overall improvement and good patient satisfaction. A combination of treatments is the key to success in most aesthetic procedures. The treatment approach needs to be in alignment with patient goals which in this case was debulking of the lower face. In some cases, masseter hypertrophy could be an issue; Botulinum toxin may also be a treatment of choice to reduce the bulk of the muscle. In conclusion, to effectively address lower face ageing, a holistic, individualistic approach with a combination of energy-based devices, PCLA threads, and HA fillers help in lifting the sagging tissue, contouring the face, and producing an overall improvement in the skin.
